# The Sufferings of Christ

**Published:** 1887-10-15

**Authors:** 


### WORDS OF CONSOLATION.

[Communications for this column should be addressed, "Chaplain," care of the Editor of THE HOSPITAL, who will gratefully welcome any suggestions or inquiries from matrons, nurses, and the staff generally.]

### LIV.—The Sufferings of Christ.

*(For Reading to the Sick.)*

As we are told so often in the accounts of the Passion that Jesus held His peace, or that He answered nothing, there would seem some very strong reason for His silence over and above the meekness with which He endured provocation. We shall observe, as we continue, how it appears that He suffered not only for sin as a whole, but that He suffered also in all His members for the separate sins we have committed. He seems to have made Atonement in detail for every class of sin. He endured mental agony for our bad thoughts, mind suffered for mind. He suffered in the body for the sins of our bodies, and in the members for the sins of our members. His hands were tied and nailed and pierced for the sins our hands have committed. His feet were fixed and pierced for the sins our feet have hurried us to commit. We shall allude to these aspects of the Cross again. Meanwhile may we not notice in the long, patient, protracted silence under grievous provocation, the Atonement made for the multitude of sins which our tongues have committed? Let us but call to mind a few occasions when we have spoken unadvisedly with our lips, or caused pain to others by saying unkind and wicked things; and does it not seem as if some special sacrifice were required to atone for the misuse of the most unruly member we possess? But besides suffering for us in detail, He has left us an example at each point of the sacrifice that we should follow in His steps. People who are repenting of sins of the tongue must often be found in the company of Jesus in respect of silence. This is a necessary part of amendment. They have to learn how to endure in meekness and silence hard words or reproaches which they would once have returned. Or, if they cannot always be silent under provocation, they have at least to show that they will take no vengeance in language, and will only give gentle answers back again. How many of us, alas! have had, or still have, a way, when spoken to unfairly, of answering back in the same strain. We have thought it unjust to ourselves for anyone to pass a disparaging remark, and not to let them have our "say" in return. To be provoked, and yet to remain silent; to be spoken against and to hold our peace; to hear what we dislike being said and not to reply! Ah, there are many of our acquaintance who would marvel greatly, as Pilate did, if we were to try and be as like Jesus as this. Silence under provocation is one great lesson of that long night's endurance. All true repentance is based upon love of the Atonement. If in your case the tongue has proved the most unruly member, and you would now steadily persevere until you have learnt to govern it, you must often look back at your Saviour, reviled, yet reviling not again. You must also believe, what is so difficult at the very time such faith is most needed, that you really have the *best* of your adversary by keeping silence. The most powerful people in the world are those who govern the tongue. "Blessed are the meek, for they shall inherit the earth" (Matt. v, 5).

*(To be continued.)*

